# Mutation-induced perturbation of the special pair P840 in the homodimeric reaction center in green sulfur bacteria

**DOI:** 10.1038/srep19878

**Published:** 2016-01-25

**Authors:** Chihiro Azai, Yuko Sano, Yuki Kato, Takumi Noguchi, Hirozo Oh-oka

**Affiliations:** 1 Division of Material Science (Physics), Graduate School of Science, Nagoya University, Nagoya 464-8602, Japan; 2 Department of Biological Sciences, Graduate School of Science, Osaka University, Toyonaka, Osaka 560-0043, Japan

## Abstract

Homodimeric photosynthetic reaction centers (RCs) in green sulfur bacteria and heliobacteria are functional homologs of Photosystem (PS) I in oxygenic phototrophs. They show unique features in their electron transfer reactions; however, detailed structural information has not been available so far. We mutated PscA-Leu688 and PscA-Val689 to cysteine residues in the green sulfur bacterium *Chlorobaculum tepidum*; these residues were predicted to interact with the special pair P840, based on sequence comparison with PS I. Spectroelectrochemical measurements showed that the L688C and V689C mutations altered a near-infrared difference spectrum upon P840 oxidation, as well as the redox potential of P840. Light-induced Fourier transform infrared difference measurements showed that the L688C mutation induced a differential signal of the S-H stretching vibration in the P840^+^/P840 spectrum, as reported in P800^+^/P800 difference spectrum in a heliobacterial RC. Spectral changes in the 13^1^-keto C=O region, caused by both mutations, revealed corresponding changes in the electronic structure of P840 and in the hydrogen-bonding interaction at the 13^1^-keto C=O group. These results suggest that there is a common spatial configuration around the special pair sites among type 1 RCs. The data also provided evidence that P840 has a symmetric electronic structure, as expected from a homodimeric RC.

The primary process of photosynthesis consists of a series of light-induced electron transfer (ET) reactions within a pigment-associated membrane-spanning protein complex called a photosynthetic reaction center (RC)^1^. In the RC, solar light induces a charge separation between a special pair of (bacterio)chlorophylls ((B)Chls) (P) and a primary acceptor (A_0_); then, the electron is consecutively transferred to electron acceptors aligned in order on the ET pathway. RCs are classified into 2 groups, type 1 and type 2, based on their functional similarity to Photosystem (PS) I and PS II, respectively, in oxygenic phototrophs^2^. In type 1 RCs, A_0_ is chlorophyll (Chl) *a*, and the terminal electron acceptor is a [4Fe–4S] cluster. In type 2 RCs, A_0_ is (bacterio)pheophytin, and the terminal electron acceptor is a quinone molecule. PS I and PS II form a linear electron transport chain from water to NADPH in oxygenic phototrophs, whereas only one of the types of RC functions in anoxygenic phototrophs. The type 1 RCs are found in green sulfur bacteria^3^, heliobacteria^4^,^5^, and phototrophic acidobacteria^6^, and the type 2 RCs are in purple bacteria and filamentous anoxygenic phototrophs^7^.

High-resolution X-ray structures were first reported for the type 2 RCs in purple bacteria (PbRC)^8^, and subsequently for PS I^9^,^10^ and PS II^11^,^12^,^13^. These RCs are known as heterodimeric RCs and consist of 2 highly homologous but not identical polypeptides. The 2 polypeptides are folded in pairs so as to make up very similar spatial configurations of transmembrane α-helices, thus providing a highly symmetrical arrangement of redox cofactors^1^. In contrast, type 1 RCs in anoxygenic phototrophs are homodimers; it is thought that they subsequently diverged into heterodimeric RCs^14^,^15^. The homodimeric RCs in green sulfur bacteria (GbRC) and heliobacteria (HbRC) have thus been targets for structural and functional studies to understand the nature of a putative ancestral RC^16^,^17^,^18^. Although the molecular species of the redox cofactors in these RCs are virtually identical to those of PS I, their spatial arrangements and respective ET reactions seem to be unique and significantly different from those in PS I^3^,^4^,^5^,^6^.

The special pairs in GbRC and HbRC have a dimeric structure consisting of BChl *a* (P840)^3^ and BChl *g* (P800)^4^,^5^, respectively. Their midpoint redox potentials (*E*_m_s), +240 mV for P840^3^ and +225 mV for P800^4^,^5^, are much lower than the *E*_m_ of P700 in PS I (Chl *a*/*a*′ dimer) and that of P870 in PbRC (BChl *a*/*a* dimer), which both had an *E*_m_ of ~+500 mV^19^. The mechanism underlying such extremely low *E*_m_s is not yet known. One of the factors determining the *E*_m_ of the special pair is the charge distribution on a (B)Chl dimer^20^; this distribution is determined by the symmetry of the dimer, as well as its protein environment. However, whether the structures and reactions of the homodimeric RCs are truly symmetric is still unknown^3^,^21^. Asymmetry could be introduced by the assembly of 2 identical RC polypeptides in an asymmetrical way or the asymmetrical binding of peripheral subunits such as membrane-bound cytochrome *c* (cyt *c*_*z*_ in green sulfur bacteria) and the F_A_/F_B_ protein.

Light-induced Fourier transform infrared (FTIR) difference spectroscopy has been used to study the homodimeric GbRC and HbRC to investigate the molecular interactions of the special pairs at the atomic level^21^,^22^,^23^,^24^,^25^,^26^. The P840^+^/P840 FTIR difference spectrum for GbRC showed 2 prominent bands for P840^+^; these bands were assigned to the 13^1^-keto C=O stretching vibrations of constituent BChl *a* molecules^21^,^26^, and their presence implied that there is an asymmetric charge distribution, at least on the infrared (IR) timescale determined by the uncertainty principle (i.e., subpicsecond). In contrast, the P800^+^/P800 FTIR difference spectrum for HbRC showed a single positive 13^1^-keto C=O signal; this observation suggests that the positive charge is significantly delocalized over the BChl *g* dimer on the IR timescale^21^,^25^. Another prominent feature of the P800^+^/P800 difference spectrum was a differential signal arising from the S-H stretching vibration of an H-bonded cysteine side chain^21^,^25^. PshA-Cys601, which was predicted to be located near the histidine ligand of P800, was proposed to be responsible for this S-H signal. However, the molecular interactions of P840 and P800 with surrounding proteins remain unclear, because X-ray crystallographic structures of the homodimeric RCs have not been available to date. It is also unknown how reliable the structural models of the homodimeric RCs are when they are constructed just from the X-ray structure of PS I based on the amino acid sequence alignments of their RC core polypeptides, because their sequence similarity is significantly low (identity <15%)^18^.

Recently, a novel technique for site-directed mutagenesis of the homodimeric GbRC, which was designated the ‘*pscA* gene duplication method’^16^, has been developed in the thermophilic green sulfur bacterium *Chlorobaculum tepidum*. This method duplicates the *pscA* gene coding for the RC core polypeptide by introducing another mutated *pscA* gene into the *recA* gene locus. Any mutant which would bear serious defect in the modified RC could thus survive, since the authentic *pscA* gene ensures the expression of the wild-type (WT) RC. Furthermore, different tags attached to the authentic (Strep-tag) and mutated (6xHis-tag) *pscA* genes, respectively, enable the isolation of 3 kinds of RC complexes, that is, Strep/Strep-tag WT RC, Strep/His-tag heterodimeric mutant RC, and His/His-tagged homodimeric mutant RC, using tandem affinity purification procedures (for details of the strategy, see ref. 16). In this study, using this *pscA* gene duplication method, we introduced site-directed mutations into the GbRC protein to modify the local structure around P840. The effects of mutations on the structures and properties of P840 were investigated by spectroelectrochemistry and light-induced FTIR difference spectroscopy. The structural homology around the special pair sites among type 1 RCs, together with the electronic structure of P840 in the homodimeric GbRCs, is discussed.

## Results

### Site-Directed Mutagenesis of the Amino Acid Residues Located Close to P840

**Figure 1:**
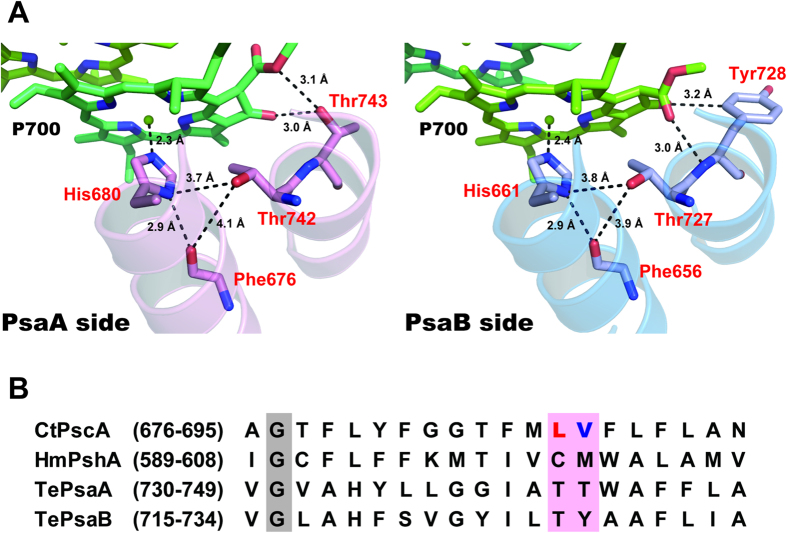
Molecular interactions of P700 in PS I, and sequence comparison of the nearby residues between different type 1 RCs. (**A**) The X-ray structure of cyanobacterial PS I, revealing the interactions of P700 (PDB ID: 1JB0^9^). The left and right panels are the views from PsaA and PsaB, respectively. Chl *a*′ is colored in green, while Chl *a* is in yellow-green. (**B**) Amino acid sequence alignments of the core polypeptides of type 1 RCs around the residues interacting with the special pair Chl. Leu688 and Val689 of PscA are colored in red and blue, respectively, while the corresponding residues in the polypeptides of type 1 RCs are shaded in pink. The gray-shaded residues are completely conserved in all the core polypeptides of type 1 RCs. The numbers in parentheses indicate the positions of the amino acid residues in the alignments. CtPscA, *Chlorobaculum tepidum* PscA; HmPshA, *Heliobacterium modesticaldum* PshA; TePsaA/B, *Thermosynechococcus elongatus* PsaA/B.

To identify amino acid residues that are predicted to interact with P840 in the GbRC, the amino acid sequence of the core polypeptide of GbRC, PscA, was aligned with those of other type 1 RCs, that is, PshA of HbRC, and PsaA/B of PS I. The geometry of each amino acid position was estimated from the X-ray structure of cyanobacterial PS I (PDB ID: 1JB0^9^; Fig. 1). In the case of PS I, the Chl *a*/*a*′ heterodimer in P700 is associated with the heterodimeric PsaA/B proteins with asymmetric H-bond interactions (Fig. 1A). The 13^1^-keto C=O group in Chl *a*′ is H-bonded with the OH group of the conserved PsaA-Thr743, whereas that in Chl *a* does not form an H-bond to any residue in PsaB^27^,^28^. These asymmetric H-bond interactions are thought to be the source of the split 13^1^-keto C=O bands in the P700^+^/P700 FTIR difference spectrum^22^. The corresponding residues in GbRC and HbRC are the non-H-bonding residues PscA-Val689 and PshA-Met602, respectively (Fig. 1B); this observation is consistent with previous spectroscopic observations that the 13^1^-keto C=O groups in P840 and P800 are free from H-bonding^21^,^26^,^29^. In addition, each of the 2 axial His ligands in P700 (PsaA-His680 and PsaB-His661) seems to interact with a nearby threonine residue (PsaA-Thr742 and PsaB-Thr727, respectively) through an H-bond to the main chain amide C=O (PsaA-Phe676 and PsaB-Phe656) (Fig. 1A). The corresponding residues in GbRC and HbRC are PscA-Leu688 and PshA-Cys601, respectively (Fig. 1B); this result agrees well with the observations of a characteristic cysteine S-H signal in the P800^+^/P800 FTIR difference spectrum, and the absence of such a signal in the P840^+^/P840 spectrum^25^. Therefore, PscA-Leu688 and PscA-Val689 in GbRC are expected to be located near P840 and affect its physicochemical properties. These 2 amino acid residues thus were feasible targets for the present mutagenesis study.

To introduce H-bond interactions with P840 in the homodimeric GbRC, PscA-Leu688 and PscA-Val689 were substituted with cysteine residues by using the genetic tools that we have recently developed in *C. tepidum*^16^,^30^. These mutations, L688C and V689C, respectively, especially the former, were designed to mimic the interaction of PshA-Cys601 with P800 in HbRC. The merit of intorducing a cysteine residue is that a desired modification, if obtained, could be detected by monitoring the characteristic S-H signal of a cysteine side chain around 2550 cm^−1^, where no signals of other amino acids as well as chromophores interfere, in FTIR analysis. According to the *pscA* duplication method^16^, the L688C and V689C RCs were constructed by expressing the mutated PscA with an N-terminal hexahistidine tag (6xHis-tag) in the *C. tepidum* SA-∆*recA* strain, which expresses the non-mutated PscA with an N-terminal strep-tag II (Strep-tag) (for details, see Text S1). The His/His-tagged homodimeric mutant RCs could be obtained as flow-through fractions in the second Strep-tag affinity chromatography after recoveries of adsorbed fractions in the first His-tag affinity chromatography. The His/Strep-tagged heterodimeric mutant RCs were also obtained as adsorbed fractions in the second chromatography; their yields were, however, only less than 5% of the amounts of the His/His-tagged homodimeric RCs. In the present study, we thus focused on the His/His-tagged homodimeric mutant RCs for further analyses. The His/His-tagged WT RC consisting of a pair of a non-mutated PscA with an N-terminal 6xHis-tag was used as a WT control.

The absorption spectra of the His/His-tagged homodimeric mutant RCs were virtually identical to the spectrum of the previously reported *C. tepidum* RC^3^,^16^, except that the L688C RC showed a slight blue shift of the Q_y_ absorption peak and an intensity decrease of the shoulder at 840 nm (see Supplementary Fig. S1 online). In the presence of 60% glycerol, both the L688C RC and the V689C RC showed light-induced charge separation activity comparable to that shown by the WT RC (see Supplementary Fig. S2 online). Under this condition, the ET reaction from cyt *c*_z_ to P840 was completely inhibited via the prevention of a conformational fluctuation of cyt *c*_z_. The decay kinetics of the photooxidized P840^+^ of individual RCs were well fitted with single exponential functions with following time constants: 77 ms for the WT RC, 73 ms for the L688C RC, and 68 ms for the V689C RC. This relaxation can be attributed to charge recombination between P840^+^ and reduced FeS clusters^3^,^16^. Thus, both the homodimeric mutant RCs were fully functional and retained all the redox cofactors used by the WT RC. Meanwhile, the recombination rate was slightly faster in both the mutant RCs, implying some perturbations of the redox property of P840 by the mutations.

### Spectroelectrochemical Analysis of P840

**Figure 2:**
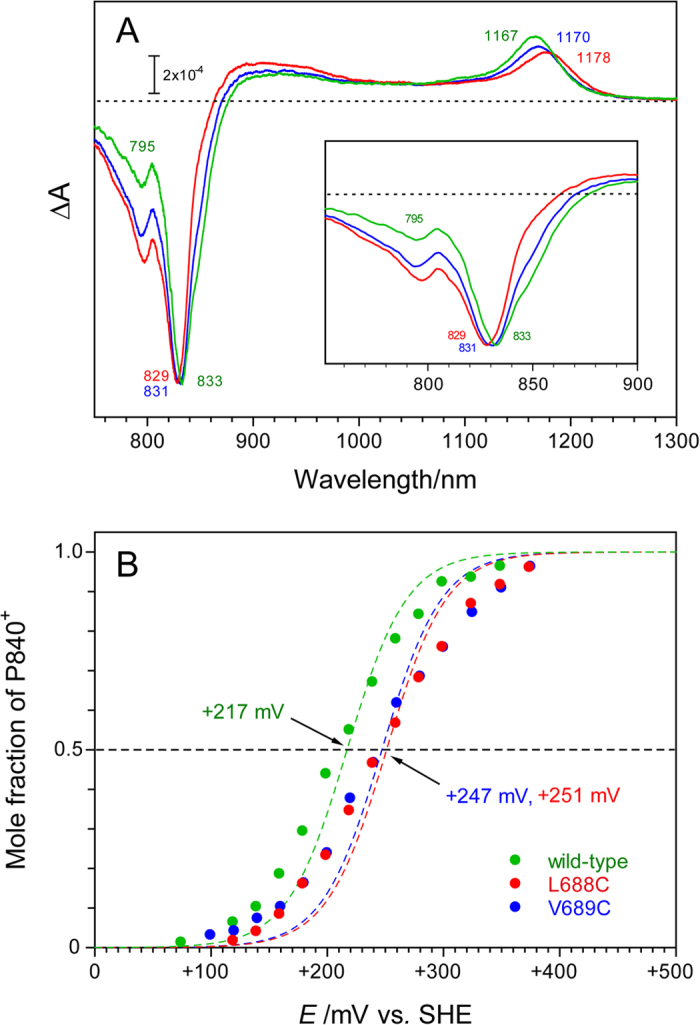
Electrochemically induced near-IR difference spectra of P840 (A) and Nernst plots of its redox reaction (B) in the wild-type (green line), the L688C (red line) and the V689C (blue line) RCs at 297 K. (**A**) P840 was oxidized by setting the working electrode at +400 mV and was subsequently reduced at −50 mV in the OTTLE cell. The inset shows expanded spectra in the region of 750–900 nm. (**B**) The mole fraction of P840^+^, which was calculated from the difference absorption changes at the positive peak around 1170 nm in the near-infrared difference spectra, was plotted against potentials of the working electrode. Numerical numbers with arrows represent the empirical *E*_m_ values estimated from least-squares fitting of the plots with theoretical Nernst curves (colored dashed lines) for one-electron redox reactions.

The effects of the L688C and the V689C mutations on the electronic structure of P840 were analyzed by measuring electrochemically induced difference spectra in the near-IR region (Fig. 2A). The P840^+^/P840 difference spectrum of the WT RC showed 3 characteristic absorption peaks, as previously reported^16^,^26^: a small negative peak at 795 nm, a large negative peak at 833 nm accompanied by a shoulder around 850 nm, and a broad positive peak at 1167 nm. Both the L688C and the V689C mutations altered these peaks (data summarized in Table 1). The decrease in intensity of the 850 nm shoulder, especially in the L688C RC, was the most noteworthy (Fig. 2A, inset); this RC also demonstrated a slight blue shift of the 833 nm peak. Since the absorption band specific to P840 is the 850 nm shoulder rather than the 833 nm peak in the WT RC^3^,^31^, the intensity decrease at 850 nm directly indicates the perturbation of the special pair interaction by these mutations. The positive peak at 1167 nm, attributed to the absorption of the radical cation of P840^32^,^33^,^34^, was red-shifted by 11 nm and 3 nm by the L688C and V689C mutations, respectively; this observation implies that these mutations caused a change in the electronic structure of P840^+^.

The *E*_m_ value of the P840^+^/P840 couple was determined from the Nernst plot of the intensity of the positive peak of P840^+^ around 1167 nm (Fig. 2B). The *E*_m_ value of +217 mV obtained for the WT RC is close to the values reported previously^35^,^36^,^37^. The L688C and V689C mutations similarly upshifted the *E*_m_ by about 30 mV, resulting in values of +251 mV and +247 mV, respectively (Table 1). This effect on the *E*_m_(P840^+^/P840) also indicates that PscA-Leu688 and PscA-Val689 are located near P840 and that their cysteine substitutions altered the electronic structure of P840.

### Light-Induced FTIR Analysis of P840

**Figure 3:**
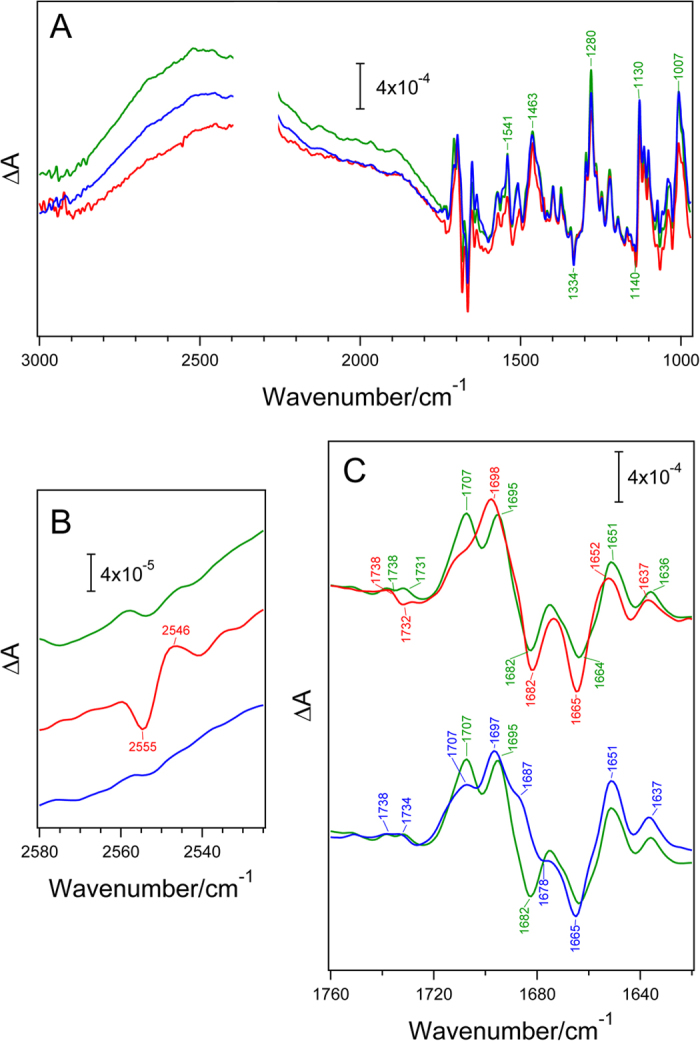
Light-induced P840^+^/P840 FTIR difference spectra of the wild-type (green line), the L688C (red line) and the V689C (blue line) RCs at 220 K. (**A**) Whole region (3000–970 cm^−1^); (**B**) S-H stretching region (2580–2525 cm^−1^); (C) C=O stretching region (1760–1620 cm^−1^). The spectra in the 2400–2300 cm^−1^ regions are not shown because of the large contribution of CO_2_ bands.

The effects of the L688C and V689C mutations on P840 were studied in more detail using light-induced FTIR difference spectroscopy. Figure 3A shows the overall P840^+^/P840 FTIR difference spectra. In the WT RC, a broad intervalence band around 2500 cm^−1 ^and strong phase-phonon lines at 1541, 1463, 1280, 1130, and 1007 cm^−1^ (the expanded spectra in the 1800–970 cm^−1^ region are presented in Supplementary Fig. S3 online) were observed as reported previously^21^,^24^,^26^; these results indicate the presence of charge delocalization over the 2 BChls *a* on P840^+^^21^,^24^,^26^,^38^,^39^. In the L688C and V689C RCs, the overall spectral features were similar to those of the WT RC, although the position of the broad intervalence band was slightly downshifted with some intensity changes; this result suggests that the mutations caused some modification in the electronic structure of P840^+^. The effects were slightly larger in the L688C RC than the V689C RC, which is consistent with results of the near-IR bands for P840 and P840^+^ (Fig. 2A).

In the L688C RC, a small differential signal was observed at 2555(−)/2546(+) cm^−1^ on the broad intervalence band (Fig. 3B). This signal is very similar to the 2560(−)/2550(+) cm^−1^ bands in the P800^+^/P800 FTIR difference spectrum of *Heliobacterium modesticaldum*; these bands were assigned to the S-H stretching vibration of PshA-Cys601 located near the histidine ligand of P800 21,25. Thus, the 2555/2546 cm^−1^ bands in the L688C RC spectrum most probably arise from the S-H vibration of PscA-Cys688, which was introduced to the PscA of *C. tepidum* at the position corresponding to the PshA-Cys601 of *H. modesticaldum*. The appearance of the similar S-H bands indicates that the interaction of PshA-Cys601 with P800 in HbRC was reproduced in the L688C RC in *C. tepidum*, although the 4–5 cm^−1^ lower frequencies in the latter suggest the presence of a slightly stronger H-bond interaction in the PscA-Cys688 S-H. It is noteworthy that no split of the band was observed, as with the S-H signal in the P800^+^/P800 spectrum^25^, in spite of the fact that there are 2 cysteine residues around P840 in the homodimeric RC. In contrast, there was no obvious signal attributable to the S-H band in the spectrum for the V689C RC or the WT RC (Fig. 3B).

The L688C and V689C mutations induced specific changes in the 1710–1680 cm^−1^ region of the 13^1^-keto C=O stretching vibrations of BChl *a*^21^,^26^ (Fig. 3C). In the spectrum for the WT RC, a negative peak at 1682 cm^−1^ and 2 prominent positive peaks at 1707 and 1695 cm^−1^ were assigned to the 13^1^- keto C=O bands of P840 and P840^+^, respectively (Fig. 3C, green lines)^21^,^26^. It is noted that the strong negative band at 1664 cm^−1^ has no contribution of the 13^1^- keto C=O vibration as shown by the previous FTIR measurement upon P840 triplet formation^23^ and probably arises from the amide I vibration (C=O stretch of backbone amide). In the spectrum for the L688C RC, one strong positive peak appeared at 1698 cm^−1^ ascribable to the 13^1^-keto C=O of P840^+^ with a remaining weak shoulder around 1707 cm^−1^, whereas the negative peak at 1682 cm^−1^ due to the 13^1^-keto C=O of P840 was virtually unaffected (Fig. 3C, red line). In contrast, in the spectrum for the V689C RC, the negative 13^1^-keto C=O band at 1682 cm^−1^, present in the spectrum for the WT RC, seemed to have downshifted to 1678 cm^−1^, while a positive band appeared at 1697 cm^−1^, along with shoulders at 1707 cm^−1^ and 1687 cm^−1^ (Fig. 3C, blue line). These changes in the 13^1^-keto C=O bands in the P840^+^/P840 spectra indicate that significant perturbations in the H-bond interaction at the keto C=O and/or in the charge distribution on the BChl *a* dimer in P840^+^ were induced by the L688C and V689C mutations.

## Discussion

Although the natural transformation system of *C. tepidum* was established in 2001^40^, there have been no site-directed mutagenesis studies on the homodimeric RC since, except for one on the deletion mutant of a peripheral subunit, PscD^41^. This lack of studies is mainly because almost all mutations that might affect RC activity would inevitably cause serious growth defects in the photoautotrophic *C. tepidum*^16^. However, by using the *pscA* gene duplication method, we were able to construct and isolate 2 site-directed mutants of the homodimeric GbRC: PscA-Leu688 and PscA-Val689 predicted to be located near P840, based on the X-ray structure of PS I, were substituted with cysteine (Fig. 1). Both the L688C and V689C mutations caused the desired effects on the results of various measurements: an upshift of the *E*_m_(P840^+^/P840) by about 30 mV (Table 1 and Fig. 2B), a decrease in the intensity of the Q_y_ band for P840 at ~850 nm, a redshift of the P840^+^ band at ~1170 nm in the electrochemically induced near-IR difference spectra (Fig. 2A), a downshift of the intervalence band around 2500 cm^−1^, and changes in the vibrational bands in the keto C=O and S-H stretching regions in light-induced FTIR difference spectra (Fig. 3). These data clearly demonstrate that the *pscA* gene duplication method^16^ is a highly practical technique for introducing mutations into the homodimeric GbRC.

Meanwhile, only small amounts of the His/Strep-tagged heterodimeric mutant RCs hindered further spectroscopic analyses in the present study. We consider that a combination of two different factors would have induced these extremely low yields. First, the expression level of the *6xhis-psc*A gene on the plasmid would be much higher (about 4- to 5-fold) than that of the strep-*pscA* gene on a chromosome, simply reflecting their copy numbers of about 9–12 for plasmid and 2–4 for chromosome, respectively, in *C. tepidum*^30^. Therefore, the probability of the dimerization between the His-tagged and Strep-tagged PscA polypeptides must be low. Second, the RC with two different N-terminal structures (i.e., His/Strep-tags) would be less stable than the RC with two identical structures (i.e., His/His-tags), even if the second *pscA* gene is not mutated. In fact, the amount of the non/His-tagged WT RC, which had a single His-tag rather than two different tags, has been estimated to be about one-fifth compared to that of the His/His-tagged WT RC by LC-MS/MS analysis, when the authentic and the second *6xhis-pscA* (i.e., not mutated *pscA*) genes were expressed together on a chromosome^16^. Nevertheless, biochemical and spectroscopic analyses of the His/Strep-tagged heterodimeric mutant RCs are still in progress, along with an effort to improve their low yields as much as possible by modifying the strategy a little.

The L688C and V689C mutations in the His/His-tagged homodimeric mutant RCs upshifted *E*_m_(P840^+^/P840) by about 30 mV (Fig. 2B), increasing the free energy gap between the neutral P840FeS and charge separated P840^+^FeS^−^ states. This result appears to be consistent with the observation that the relaxation rate of the P840^+^FeS^−^ state was slightly accelerated in the L688C and V689C RCs (see Supplementary Fig. S2 online). The charge recombination rates involving the terminal electron acceptor FeS are known to be much faster than that expected from the empirical distance-rate relationship^42^,^43^ using the geometry of PS I, where the edge-to-edge distance between P700 and FeS is longer than 30 Å. This implies that the electron transfer does not occur in single-step tunneling through insulating protein but may occur in multistep tunneling^42^,^43^. In fact, in PS I, the relaxation of the P700^+^FeS^−^ state is considered to occur through thermally activated repopulation of the P700^+^A_1_^−^ state at room temperature^44^, and hence the rate-limiting step should be the reverse electron transfer from FeS^−^ to A_1_^44^. If the charge recombination of P840^+^FeS^−^ in GbRC also occurs through the P840^+^A_1_^−^ state, the P840^+^FeS^−^ relaxation rate would be more dependent on the redox property of A_1_, including its surrounding nature, than that of P840. This could be the reason for the relatively small effect of the *E*_m_ change of P840 on the recombination rate of P840^+^FeS^−^ in the mutant RCs. However, the further evaluation for the mutation effect should await detailed structural information on the GbRC.

**Figure 4:**
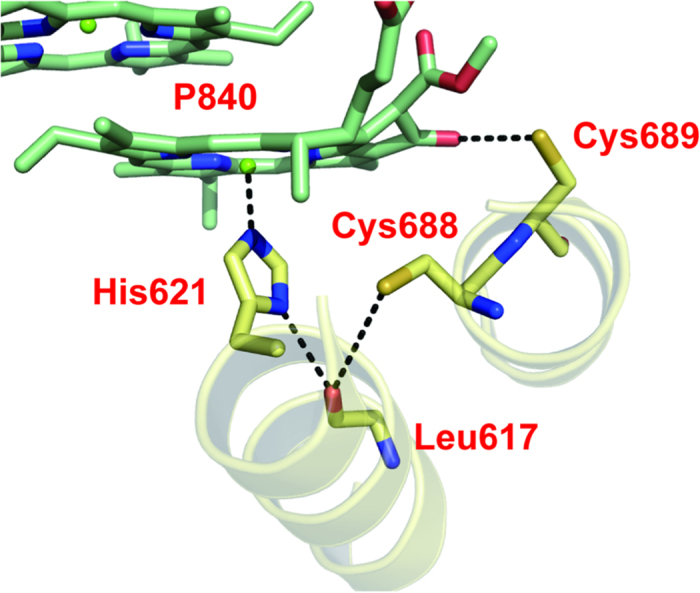
Structural model of an H-bond network around P840 in the L688C and the V689C RCs. The structural model was constructed with the mutated PsaA in which Thr-742 and Thr-743 were substituted with cysteine residues, to show the artificially introduced H-bond network in the L688C and V689C RCs. The modelling was performed by the homology modelling program MODELLER (ver. 9.15^50^), using the X-ray structure of PS I (PDB ID: 1JB0^9^) as a template.

The FTIR difference spectrum for the L688C RC, in which PscA-Leu688 in GbRC was replaced with a cysteine residue mimicking PshA-Cys601 in HbRC (Fig. 1), had a differential S-H signal at 2555(−)/2546(+) cm^−1^ and a single prominent band at 1698 cm^−1^ ascribable to the 13^1^-keto C=O vibration of P840^+^ (Fig. 3, red lines). These characteristics are significantly different from those in the WT GbRC spectrum, in which there was no S-H signal, and 2 keto C=O bands were observed at 1707 cm^−1^ and 1695 cm^−1^, respectively (Fig. 3, green lines). However, these characteristics are very similar to those of the HbRC spectrum, in which there were an S-H signal at 2560(−)/2550(+) cm^−1^ and a single 13^1^-keto C=O band of P800^+^ at 1702 cm^−1^ 21,25. This observation indicates that the newly introduced cysteine at the 688 position in PscA interacts with P840 in a manner similar to the way that PshA-Cys601 interacts with P800 in HbRC. In the sequence alignment of the core polypeptides of type 1 RCs (Fig. 1), PscA-Leu688 of GbRC and PshA-Cys601 of HbRC correspond to PsaA-Thr742/PsaB-Thr727 of PS I. According to the X-ray structure of PS I^9^, the oxygen atoms of the PsaA-Thr742 and PsaB-Thr727 side chains are located close to the N_δ_ atoms of the histidine ligands of P700 (O··N distance is 3.73–3.81 Å) and the oxygen of the main chain C=O (O··O distance is 3.85–4.13 Å) on the PsaA and PsaB sides, respectively (Fig. 1). However, because the N_δ_ site of the histidine is probably protonated and H-bonded to the main chain C=O (N··O distance is 2.91–2.92 Å) (Fig. 1), it may be difficult for this histidine to accept another H-bond. Thus, we speculate that the S-H of PscA-Cys688 in the L688C RC provides an H-bond to the main chain C=O, which simultaneously forms an H-bond with the histidine ligand (Fig. 4); this would also be the case for HbRC. The downshift of the S-H band upon P840^+^ (Fig. 3B) and P800^+^ 25 formation may be caused by either the electrostatic effect on the S-H vibration by the positive charge formation on the BChl dimer or by an influence on the H-bond interaction through the histidine ligand.

Generally, in FTIR difference spectra, upon cation formation on a special pair (B)Chl in RCs, the frequencies of negative 13^1^-keto C=O peaks of the neutral state directly reflect their H-bonded interactions. In contrast, positive 13^1^-keto C=O peaks due to the cation state reflect the extent of charge delocalization over the (B)Chl dimer on the timescale of IR spectroscopy (i.e., subpicosecond) as well as the strengths of H-bond interactions^21^,^45^. The 2 separate 13^1^-keto C=O bands at 1707 and 1695 cm^−1^ in P840^+^ of the WT GbRC (Fig. 3C, green line) were first interpreted as originating from either asymmetric interactions at the two 13^1^-keto C=O groups in P840 or asymmetric charge distribution over the BChl *a* dimer on the subpicosecond timescale with charge exchange between the 2 BChl *a* molecules on a much slower timescale^26^. However, the latter interpretation was favored later^21^ based on the observation that a single differential signal of the 13^1^-keto C=O was detected at 1674/1685 cm^−1^ in a ^3^P840/P840 difference spectrum, which suggested a symmetric interaction of the keto C=O^23^. In contrast, the presence of a single positive 13^1^-keto C=O band at 1702 cm^−1^ in the P800^+^/P800 FTIR spectrum for HbRC indicated significant delocalization of the positive charge over the BChl *g* dimer, with symmetric interactions in the subpicosecond timescale^21^,^25^. Introduction of the S-H interaction in the L688C mutant RC, which is analogous to HbRC, changed the split signals at 1707 cm^−1^ and 1695 cm^−1 ^to a single strong signal at 1698 cm^−1^ without changing the negative 13^1^-keto C=O band at 1682 cm^−1^ (Fig. 3C). The absence of the change in the 1682 cm^−1^ band of P840 confirmed that the H-bond interaction at the 13^1^-keto C=O did not change by this mutation as expected, and hence the large change in the positive bands of P840^+^ should arise from the change in the charge distribution over the dimer on the subpicosecond timescale. Thus, it is suggested that the introduced S-H interaction in the L688C RC made the charge transfer between the 2 BChl *a* molecules faster than this timescale resulting in an even charge distribution. The cation-induced 16 cm^−1 ^upshift (from 1682 cm^−1 ^to 1698 cm^−1^) in the L688C RC, half of that of monomeric BChl *a* in THF by 32 cm^−1^ 46, is also consistent with the symmetric charge delocalization over the dimer. Note that a weak shoulder left at 1707 cm^−1^ could arise from a minor component of the L688C RC that has a slow charge transfer rate possibly by a thermal fluctuation of the H-bonded structure. It is speculated that the interaction of the cysteine S-H to the main chain C=O would have changed the interaction of the histidine ligand of P840, resulting in the change in the electronic structure of the P840^+^ cation, although understanding the detail of the interaction change would require sophisticated quantum chemical calculations. The change in the electronic structure of P840^+^ is also consistent with the shifts of the near-IR band at ~1170 nm (Fig. 2A) and the intervalence band in the IR region at ~2500 cm^−1^ (Fig. 3A) in the L688C RC.

The above observations of the 13^1^-keto C=O bands of the L688C mutant also provide clear evidence that P840 has the symmetric structure of a BChl *a* dimer. If P840 had an asymmetric structure, not only the negative keto C=O band but also the positive band would be split into 2 bands even if the charge were evenly distributed. Thus, it is now clear that the split 13^1^-keto C=O bands at 1707 and 1695 cm^−1^ in P840^+^ of the WT RC does not arise from the asymmetric interactions. In addition, the absence of the band splitting in the S-H signal at 2555/2546 cm^−1^ (Fig. 3B) supports the view that the protein structure around P840 is also symmetric. The same conclusion has been obtained for P800 of HbRC by FTIR analysis^21^,^25^. Thus, it can be concluded that P840 and P800 in homodimeric RCs truly have symmetric dimer structures, although it is possible that even homodimeric RCs have asymmetric cofactor interactions via the binding of peripheral subunits in asymmetric ways. This conclusion is also consistent with previous electron paramagnetic resonance (EPR) studies, which showed a symmetric spin distribution on P840^+^ at the EPR time resolution (submicroseconds)^47^,^48^.

The cysteine S-H introduced by the V689C mutation was predicted to form an H-bond with the 13^1^-keto C=O of P840, according to the X-ray structure of PS I and the sequence alignment of type 1 RCs (Fig. 1). Indeed, the negative 13^1^-keto C=O band of P840 was clearly modified; the 1682 cm^−1 ^peak seemed to have downshifted to 1678 cm^−1^ in response to the introduction of an H-bond (Fig. 3C). The 1664 cm^−1^ band did not show a downshift but was only slightly upshifted with some intensity increase. The absence of the downshift by cysteine introduction confirmed the assignment of this band to the amide I vibration and not to the keto C=O^26^. The slight upshift and the intensity change may be attributed to the minor perturbation of polypeptide main chains induced by mutation. The 13^1^-keto C=O region of P840^+^ was also modified; 2 peaks at 1707 and 1695 cm^−1^ were changed to 3 peaks at 1707, 1697, and 1687 cm^−1^ with a strongest intensity at the 1697 cm^−1^ peak (Fig. 3C, blue line). The presence of 3 bands can be explained by considering a thermal equilibrium between 2 different H-bonded forms in P840^+^. The lowest peak at 1687 cm^−1^ clearly arises from the H-bonded 13^1^-keto C=O downshifted from 1695 cm^−1^ by 8 cm^−1^, while the highest-frequency shoulder at 1707 cm^−1^ is due to the non-H-bonded keto C=O analogous to that of the WT RC. The strongest peak at 1697 cm^−1^ can hence be attributed to the overlap of the H-bonded C=O band downshifted by 10 cm^−1^ from 1707 cm^−1^ and the non-H-bonded band that remained at 1695 cm^−1^. A similar example of the coexistence of H-bonded and non-H-bonded forms has been observed in the 13^2^-ester C=O of the bacteriopheophytin electron acceptor in PbRC^49^. The equilibrium of 2 H-bonded forms at the keto C=O in P840^+^ seems absent in neutral P840 of the V689C RC, because the original WT band at 1682 cm^−1^, which reflects a non-H-bond interaction, did not remain in the spectrum of the V689C RC. The presence of the non-H-bonded form only in P840^+^ could be caused by a C=O distance shortened upon P840 oxidation, which is reflected in the upshifted keto C=O frequencies, and hence a longer H-bonding distance between the C=O and the S-H of a newly introduced cysteine residue. In spite of the probable H-bond of the cysteine S-H to the 13^1^-keto C=O of P840, we observed no signal in the typical S-H region of 2600–2520 cm^−1^ (Fig. 3B). This observation could result from the mutual cancellation of 2 opposite shifts of the S-H band in the P840^+^ state: an upshift caused by weakening the H-bond via the shortened 13^1^-keto C=O bond, which was shown in the upshift of the keto C=O band, and a downshift due to an electrostatic effect by positive charge on P840^+^.

In conclusion, the present results demonstrate that site-directed mutations of L688C and V689C were successfully introduced into the homodimeric GbRC. The effects of these mutations, predicted from the sequence alignment of the type1 RC polypeptides, from the X-ray structure of PS I (Fig. 1), and from the previously published P800^+^/P800 FTIR spectrum for HbRC^21^,^25^, were realized as expected, providing experimental evidence for close structural relationships among the type 1 RCs of green sulfur bacteria, heliobacteria, and PS I, especially with respect to the structure around the special pair, despite the extremely low sequence homology among the core polypeptides of these RCs (identity <15%)^18^. Furthermore, FTIR analysis of the L688C mutant in comparison with the WT GbRC and HbRC showed that P840 has the structure of a symmetric BChl *a* dimer, as expected from an RC consisting of 2 identical PscA polypeptides.

## Methods

### Construction and Isolation of the Site-Directed Mutant GbRCs

Site-directed mutants of the GbRC complexes were constructed by a combined strategy utilizing the *pscA* gene duplication method^16^ and the conjugative plasmid transfer system in *C. tepidum*^30^. The host strain was the newly constructed SA-∆*recA* mutant strain (for details, see Supplementary Information online). The *6xhis-pscA* gene in pENTR-HisAB^30^ was site-specifically mutated by inverse PCR using appropriate primer sets (see supplementary Table S1 online), and the self-ligation products were cloned. The mutated 6xhis-pscA^*^B gene cluster was transferred into the conjugative vector pDSK5191^30^ and then introduced into the host *C. tepidum* cell via bacterial conjugation. The resultant *C. tepidum* mutant strain, called SA-∆*recA*/pDSK5191-6xhis-pscA^*^B, expressed the 6xHis-tag mutated PscA and the Strep-tagged WT PscA.

The His/His-tagged homodimeric mutant RC complexes (L688C RC and V689C RC) in the SA-∆*recA*/pDSK5191-6xhis-pscA^*^B cells were isolated using 2 different consecutive affinity chromatography procedures: Ni^2+^-affinity chromatography for the 6xHis-tag, and subsequent Strep-Tactin affinity chromatography for the Strep-tag (for details, see Supplementary Information online). All manipulations were performed in an anaerobic glove box (Coy Laboratory Products), where the O_2_ concentration was below 1 ppm. The His/His-tagged WT RC complex was prepared from the corresponding control strain SA-∆*recA*/pDSK5191-*6xhis-pscAB* and used as a control wild-type sample in the present study.

### Spectroelectrochemistry

An optically transparent thin-layer electrode (OTTLE) cell with an optical path length of about 180 μm was used for spectroelectrochemical measurements. An Au mesh (100 mesh per inch), a Pt black electrode, and an Ag-AgCl electrode (in saturated KCl) were used as working, counter, and reference electrodes, respectively. The electrode potential in this study is referred to a standard hydrogen electrode (SHE; +199 mV vs. Ag-AgCl). The RC preparation was suspended in buffer B (10 mM Tris-HCl [pH 8.0], and 1 mM sucrose monolaurate) containing 200 mM KCl to give an OD of ca. 0.45 at the Qy absorption peak. The following agents were employed as redox mediators: 100 μM phenazine methosulfate (*E*_m_ = +80 mV), 100 μM N,N,N′,N′-tetra-methyl-*p*-phenylene diamine (*E*_m_ = +260 mV), and 100 μM K_3_Fe(CN)_6_ (*E*_m_ = +430 mV). Absorption spectra of the samples and their changes in the OTTLE cell were measured on a spectrophotometer (JASCO, Model V670). The electrode potential was controlled using a potentiostat (Toho Technical Research, Model 2020).

### FTIR Spectroscopy

Light-induced FTIR difference spectra upon photooxidation of P840 were measured on a Bruker IFS-66/S spectrophotometer equipped with a MCT detector (D313-L), as described previously^26^. The RC preparation was desalted by repeating a 10-fold dilution with buffer B and subsequent concentration by ultrafiltration (nominal molecular weight cutoff = 100 kDa, Millipore) 3 times. The sample (OD = 20 at the Qy absorption peak, 10 μL) was lightly dried on a CaF_2_ plate (13 mm in diameter) with N_2_ gas flow, mixed with 10 μL of a 3 mM benzyl viologen solution as an exogenous electron acceptor, and dried again. The sample was then covered with another CaF_2_ plate together with 0.7 μL of a 3 mM benzyl viologen solution. The sample temperature was adjusted to 220 K in a cryostat (Oxford DN1704) with a controller (Oxford ITC-5). Difference spectra were obtained by subtraction between the 2 single-beam spectra (300 scans; 150 s accumulation for each) recorded before and after 30 s continuous-light illumination (~30 mW/cm^2^ at the sample) from a halogen lamp (Hoya-Schott HL150) equipped with a red filter (>600 nm). Spectral resolution was 4 cm^−1^.

## Additional Information

**How to cite this article**: Azai, C. *et al.* Mutation-induced perturbation of the special pair P840 in the homodimeric reaction center in green sulfur bacteria. *Sci. Rep.* **6**, 19878; doi: 10.1038/srep19878 (2016).

## Supplementary Material

Supplementary Information

## Figures and Tables

**Table 1:** Spectroelectrochemical properties of the wild-type and homodimeric mutant RCs.

RC	*E*_m_ (mV vs. SHE)	Negative peak (nm)	Positive peak (nm)
wild-type	+217	833	1167
L688C	+251	829	1178
V689C	+247	831	1170
