# Supplementary material for: Mutation-induced perturbation of the special pair P840 in the homodimeric reaction center in green sulfur bacteria
Source: Sci Rep. 2016 Jan 25;6:19878. doi: 10.1038/srep19878 (PMC4726426; doi:10.1038/srep19878)
Supplement: Supplementary Information [file srep19878-s1.doc]

# **Supplementary Information**

# **Mutation-induced perturbation of the special pair P840 in the homodimeric reaction center in green sulfur bacteria**

## Chihiro Azai, Yuko Sano, Yuki Kato, Takumi Noguchi, Hirozo Oh-oka

## **Detailed Materials and Methods**

**Bacterial Strains and Culture Conditions**

*Chlorobaculum tepidum* WT2321 [^1^](#_ENREF_1) was used as a wild-type strain in the present study. Cultivation of *C. tepidum* in liquid CL media and on solid CP plates was routinely performed in essentially the same fashion as previously described [^2^](#_ENREF_2). The growth temperature and the light intensity from incandescent lamps were set to 40°C and 30 µmol photons/m^2^/s, respectively. *Escherichia coli* DH5α [^3^](#_ENREF_3) was used for molecular cloning to construct plasmids. *E. coli* S17-1 [^4^](#_ENREF_4) was used as a donor for conjugative transfers of the pDSK5191-derivative plasmids. The *E. coli* cells were grown in liquid or on solid LB media at 37°C. The following antibiotics with specified concentrations were added to the media if required: 30 µg/mL of gentamicin, 1 µg/mL of erythromycin, or a mixture of 100 µg/mL of streptomycin and 50 µg/mL of spectinomycin for *C. tepidum*; 100 µg/mL of ampicillin, 25 µg/mL of kanamycin, 30 µg/mL of streptomycin, 100 µg/mL of spectinomycin, or 200 µg/mL of erythromycin for *E. coli*.

**Construction of *C. tepidum* SA-∆*recA* mutant strain**

The stable *C. tepidum* mutant strain SA, expressing Strep-tagged PscA polypeptides, was constructed by natural transformation [^2^](#_ENREF_2). The plasmid vector was constructed as follows: To attach the Strep-tag II [^5^](#_ENREF_5) to the N-terminus of the *C. tepidum* PscA, a 2.0-kbp fragment of the 5’-part of the *pscA* gene was excised from the pENTR-HisAB [^6^](#_ENREF_6) with *Bam*HI and *Hin*dIII, and cloned between the *Bam*HI and *Hin*dIII site of pET51b (Novagen), yielding pStrepAn51b. The 5’-part of the *strep-pscA* gene on pStrepAn51b was amplified from by PCR using the primers pscA-3561F and the T7 Promoter primer. The P*_pscA_*::*aadA* region of pUC118-P*_pscA_*::*aadA* [^6^](#_ENREF_6) was amplified by PCR using the primers pscA-4072F (*Nco*I) and dapF-4676R. These two fragments were digested with *Nco*I and ligated simultaneously into the *Sma*I site of pHP45 [^7^](#_ENREF_7), yielding pHP45-StrepA. After checking the sequence, the PCR-amplified fragment by the primers HP45-blaF and HP45-ropR was used for the DNA construct for the natural transformation. Natural transformation for construction of the SA strain was performed in the same way that the 6HA mutant strain was previously isolated [^6^](#_ENREF_6). The fully segregated allele of the streptomycin/spectinomycin-resistance clone was confirmed by the product of genomic PCR using the primers pscA-2448F and dapF-5291R and its direct DNA sequencing after growing in the liquid CL medium. To construct the SA-∆*recA* strain, the *recA* gene was disrupted in the isolated SA strain as described previously [^8^](#_ENREF_8). The primers used for the plasmid construction and analytical PCR were listed in Table S2.

**Isolation of the homodimeric mutant GbRCs**

The His/His-tagged homodimeric mutant RC complexes in the *C. tepidum* SA-∆*recA*/pDSK5191-*6xhis-pscA^*^B* strain, which expressed the 6xHis-tagged mutated PscA and the Strep-tagged wild-type PscA, were isolated using two different consecutive affinity chromatography procedures. In the first step, preparation and solubilization of crude membranes and subsequent Ni^2+^-affinity chromatography were carried out to isolate His-tagged RC complexes as described previously [^8^](#_ENREF_8). In the second step, the His/His-tagged homodimeric and His/Strep-tagged heterodimeric mutant RC complexes in the eluted fraction of the Ni^2+^-affinity chromatography were separated by the Strep-Tactin-affinity chromatography. Before the second chromatography, the eluted fraction was delated as follows: the eluted fraction was precipitated by the addition of PEG-buffer [50 mM Tris-HCl (pH8.0), 1 mM EDTA, 2 mM DTT, and 50% polyethylene glycol (PEG) 4,000] until a final concentration of PEG was reached to 12.5%. After centrifugation at 12,000×*g* for 30 min, the precipitate was dissolved in buffer A [50 mM Tris-HCl (pH8.0), 1 mM EDTA, 2 mM DTT, and 1 mM sucrose monolaurate] at a final OD of ca. 10 at the Q_y_ absorption peak. The desalted sample thus obtained was loaded onto a Strep-tactin sepharose column, in which the resin volume was about one fifth of the total volume of the sample, and the flow-through fraction containing the His/His-tagged homodimeric mutant RC was collected. The resultant RC sample was concentrated by PEG precipitation and subsequent dissolving in a small amount of buffer A as described above, and stored in an air-tight vial at -80°C until use. The contamination of the Strep-tagged wild-type PscA into the samples was estimated to be less than 1% of total amount of PscA judging from western blot analyses using specific monoclonal antibodies against the 6xHis-tag (D293-1, MBL) and the Strep-tag II (71590-3, Novagen).

## **Table S1. Primers used for site-directed mutagenesis by inverse PCR**

| Primer name | Sequence |
| --- | --- |
| L688C mutation |  |
| pscA-SD 207F (L688C) | GCAcatgaaagtaccgccaaagt |
| pscA-SD 206R | GTCttcctgttccttgccaacg |
| V689C mutation |  |
| pscA-SD 207F | GAGcatgaaagtaccgccaaagt |
| pscA-SD 206F (V689C) | TGCttcctgttccttgccaacg |

## **Table S2. Primers used for DNA constructions and analytical PCRs.**

| Primer name | Sequence | Reference ^a^ |
| --- | --- | --- |
| dapF-5291R | cacatgcatggttgacggag | [^6^](#_ENREF_6) |
| dapF-4676R | tgaggtgggcaaaccagaca | [^6^](#_ENREF_6) |
| pscA-4072F (*Nco*I) | TCAGCCATGGTATGTTCTCCTTTGTTTGAACG | [^8^](#_ENREF_8) |
| pscA-3561F | cgtcagattgtgcgtccagt | [^6^](#_ENREF_6) |
| pscA-2448F | GCTTGTCGAGTTCCTTGTAG | [^6^](#_ENREF_6) |
| HP45-blaF | CAAGGATCTTACCGCTGTTG | [^8^](#_ENREF_8) |
| HP45-ropR | GCTTACAGACAAGCTGTGAC | [^8^](#_ENREF_8) |
| T7 Promoter primer | taatacgactcactataggg |  |


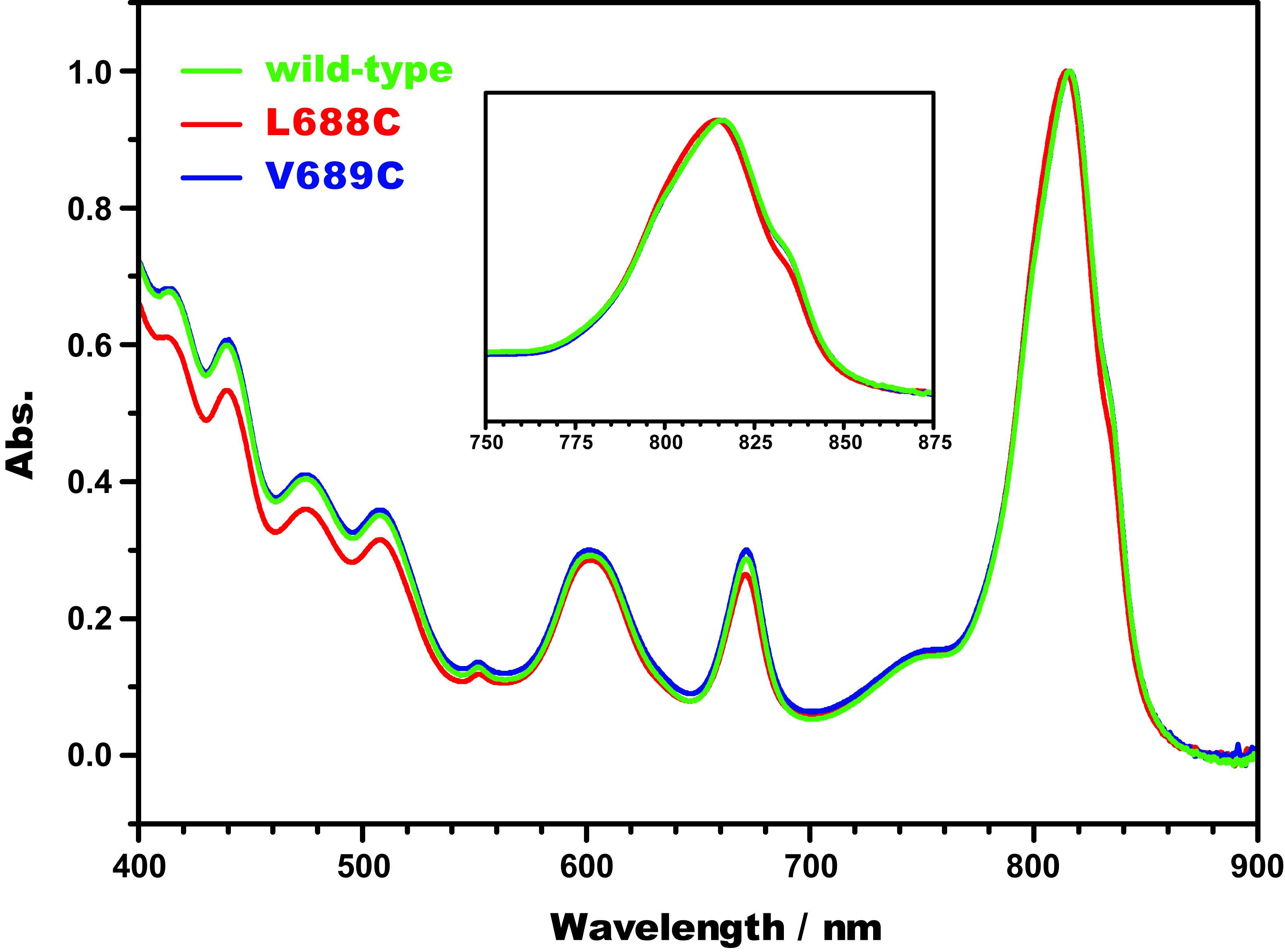


## **Figure S1. Absorption spectra of the wild-type and mutant RCs of *C. tepidum*.**

Absorption spectra were measured on a Shimadzu UVPC-3101 spectrophotometer at room temperature. The samples were diluted in an air-tight cell (optical path = 1 cm) with a buffer containing 50 mM Tris-HCl (pH 8.0), 1 mM EDTA, 2 mM DTT, 1 mM sucrose laurate, and 60%(v/v) glycerol, to give an absorbance at the Q_y_-peak (around 815 nm) of 1.0. The inset shows enlarged spectra of BChl *a* Q_y_ bands.


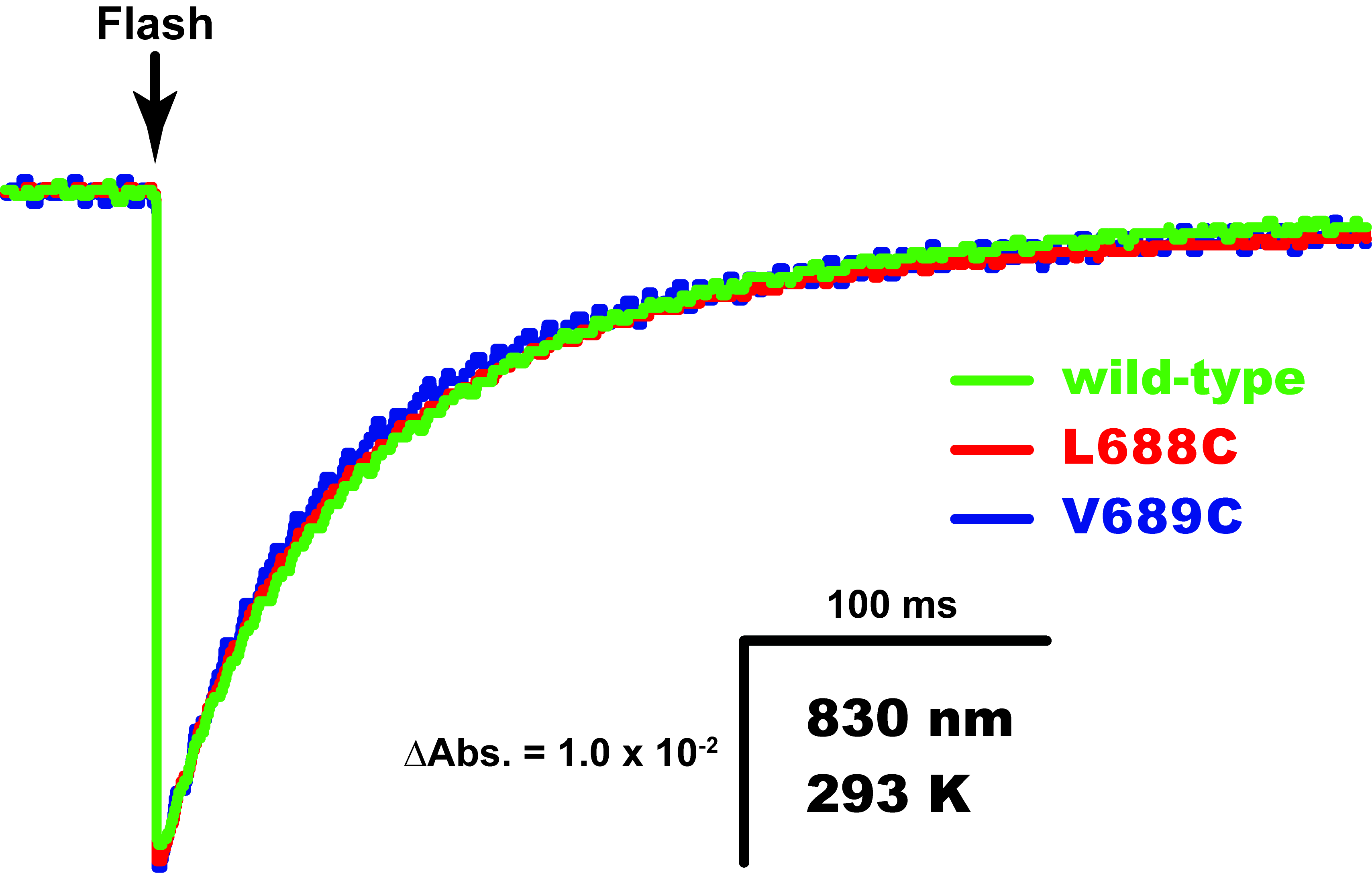


## **Figure S2. Flash-induced charge separation activities of the wild-type and mutant RCs of *C. tepidum*.**

Xe-flash-induced absorbance changes at 830 nm were measured at room temperature as described previously [^8^](#_ENREF_8). Flash duration was less than 1 ms. The samples were the same as those in Figure S1.

## **Figure S3. Light-induced P840^+^/P840 FTIR difference spectra of the wild-type and mutant RCs of *C. tepidum* in the 1800-970 cm^-1^ region.**

Green, WT; red, L688C; blue, V689C. The spectra were measured at 220 K in the presence of benzyl viologen as an electron acceptor.

**References**

1 Wahlund, T. M. & Madigan, M. T. Genetic transfer by conjugation in the thermophilic green sulfur bacterium *Chlorobium tepidum*. *J. Bacteriol.* **177**, 2583-2588 (1995).

2 Frigaard, N. U. & Bryant, D. A. Chromosomal gene inactivation in the green sulfur bacterium *Chlorobium tepidum* by natural transformation. *Appl. Environ. Microbiol.* **67**, 2538-2544 (2001).

3 Hanahan, D. Studies on transformation of Escherichia coli with plasmids. *J Mol Biol* **166**, 557-580 (1983).

4 Simon, R., Priefer, U. & Puhler, A. A Broad Host Range Mobilization System for Invivo Genetic-Engineering - Transposon Mutagenesis in Gram-Negative Bacteria. *Bio-Technology* **1**, 784-791 (1983).

5 Maier, T., Drapal, N., Thanbichler, M. & Bock, A. Strep-tag II affinity purification: an approach to study intermediates of metalloenzyme biosynthesis. *Anal. Biochem.* **259**, 68-73 (1998).

6 Azai, C., Harada, J. & Oh-oka, H. Gene expression system in green sulfur bacteria by conjugative plasmid transfer. *PLoS One* **8**, e82345 (2013).

7 Prentki, P. & Krisch, H. M. In vitro insertional mutagenesis with a selectable DNA fragment. *Gene* **29**, 303-313 (1984).

8 Azai, C. *et al.* A heterogeneous tag-attachment to the homodimeric type 1 photosynthetic reaction center core protein in the green sulfur bacterium *Chlorobaculum tepidum*. *Biochim. Biophys. Acta* **1807**, 803-812 (2011).
